# Editorial: Molecular diagnostic methods for bacteria and fungi detection

**DOI:** 10.3389/fcimb.2024.1430630

**Published:** 2024-06-04

**Authors:** Carina Almeida, Laura Cerqueira

**Affiliations:** ^1^Food Safety Unit, National Institute for Agrarian and Veterinary Research (INIAV), Vila do Conde, Portugal; ^2^LEPABE – Laboratory for Process Engineering, Environment, Biotechnology and Energy, Faculty of Engineering, University of Porto, Porto, Portugal; ^3^AliCE – Associate Laboratory in Chemical Engineering, Faculty of Engineering, University of Porto, Porto, Portugal

**Keywords:** diagnostic methods, bacteria, fungi, molecular methods, pathogens detection

In recent years, a multitude of emerging pathogens and resistance determinants have highlighted the growing urgency for rapid and multiplex testing to address a spectrum of challenges of public health, veterinary, and environmental sectors. Currently, cutting-edge technologies in molecular diagnostics have emerged as indispensable tools to effectively address: the great diversity of agents that need to be detected in different matrices, the need to provide a fast and more informative response; the need for low detection limits to allow early detection; and/or the need to produce deployable methods that can be used *in loco* or in low-resource contexts. These needs set the foundation for a broad range of molecular-based technologies that could expedite the diagnosis and provide a precise intervention.

In this Research Topic, researchers were encouraged to submit innovative works describing new molecular methodologies, or comparing existing ones, with the purpose of improving microbial detection/diagnosis. This Research Topic provides examples of diverse molecular methods, from conventional PCR, to digital PCR or isothermal amplification techniques, that proved to be good alternatives to standard culturing techniques either in terms of time to result, limit of detection or type of information obtained.

Beginning with a study on the evaluation of different methodologies; Chatelard at al. has provided a comparison of three early blood culture testing protocols in terms of their ability to shorten the time to result and implement timely/appropriate antibiotic therapy. Positive blood cultures were tested using either a multiplex PCR panel (GenMark ePlex), a combination of MRSA/SA PCR, β-Lacta and oxidase tests (multi test), or conventional identification and susceptibility testing (as the gold standard). The delay between a blood culture positivity and initial results was more pronounced in the multitest protocol. More importantly, the proportion of patients receiving appropriate antibiotic therapy within 48 h of blood sampling was higher when either multiplex PCR and multitest were implemented (90% and 88%, respectively) comparing to the conventional method (71%).

Moving to another comparison study, now on tuberculosis diagnostics, Boldi et al. has provided an important 10-year retrospective study on the performance of microscopy, PCR and culture on different respiratory specimens (sputum, induced sputum, bronchial aspirate and bronchoalveolar lavage). Culture displayed the highest sensitivity and specificity, while PCR has shown higher sensitivity and specificity than microscopy for all respiratory specimens. The diagnosis yield of bronchial aspirate was higher than that of bronchoalveolar lavage. Overall, results suggest that PCR should be systematically performed on bronchial aspirates, when available, to expedite diagnosis without significantly compromising the diagnostic accuracy.

Another subject of great relevance when dealing with significant infectious agents/diseases, such as Tuberculosis, is the availability quality control materials for appropriate proficiency testing. Early diagnosis is essential for proper treatment, especially in low- and middle-income countries that register 98% of the Tuberculosis cases worldwide, and reference materials are crucial for proper training and assessment of laboratories. Guan et al. provided us with a quality control library to be used with Xpert MTB/RIF test, a worldwide implemented assay (endorsed by the World Health Organization) that provides detection and simultaneous rifampicin resistance testing. The panel covers various probe patterns of Xpert MTB/RIF for the resistance detection. The library was constructed in two non-pathogenic bacterial strains, *E. coli* and *Bacillus subtilis*, which eliminate biosafety risks.

Still within the tuberculosis diagnostics, Owusu et al. propose a new multiplex PCR assay that allows the differentiation of *Mycobacterium tuberculosis* complex, specifically *M. tuberculosis*, *M. africanum* Lineages 5/6 and *M. bovis*, in low resource settings. No cross-reaction with other respiratory pathogens was observed and the detection limit for the different primers sets ranged between 620 and 2479 copies/ul. Validation was performed in sputum samples from 341 confirmed tuberculosis patients previously analysed by sputum smear microscopy, GeneXpert MTB/RIF assay and culture (BD BACTEC) methods. This multiplex PCR assay allowed the speciation of MTBC lineages in clinical samples, as well as the identification of mixed-lineage tuberculosis infections that can difficult treatment.

Other interesting PCR methodologies were proposed by Jiang et al. to detect two relevant fungal genera, *Aspergillus* and *Mucorales*, into paraffin-embedded tissue samples collected from patients with suspected invasive mold disease. The methodologies targeted the 18S and the 28S rRNA and presented sensitivity values ranging from 65% to 75% and specificity ranging from 82 to 97%. Interestingly, Wei et al. studied an alternative, non-invasive, approach to detect pulmonary aspergillosis (PA) without resorting to bronchoalveolar lavage fluid (BALF). They stated that the use of ultra high-performance liquid chromatography coupled with high-resolution mass spectrometry (UHPLC-HRMS) ascertain the metabolic profile of exhaled breath condensate (EBC) samples in PA patients and can be used as a non-invasive alternative method. They have identified five biomarkers for the diagnosis of PA. Compared with other methods (sputum smear and culture, BALF galactomannan assay and mNGS), the EBC method was accurate and efficient.

A promising molecular alternative in terms of sensitivity/detection limit certainly rests in digital PCR, for its ability to split the sample/reaction mix into thousands of single partitions. Tak et al. have developed an innovative droplet digital PCR (ddPCR) method to detected low number of infectious bacteria during early stages of prosthetic joint infection. The bacterial pathogen adheres and then forms microcolonies and biofilms that are very difficult to eradicate. Most often diagnosis resorts to a combined approach but depends mostly on the culture of patient-derived samples (typically tissue samples obtained during joint aspiration) which often contain very low number of cells, ending up on false negative results (due to low sensitivity). These authors make use of the ddPCR ability to provide absolute quantification of the target (without standard calibration curve), as well as its very high sensitivity, to detect early bacterial infection. They have found that ddPCR limit if detection was approximately 10 times lower than that of real-time PCR.

Within amplification-based molecular methods, isothermal techniques have evolved very quickly in the last years, especially due to the pressure of the SARS-CoV-2 pandemic crisis that boosted the development of point-of-need solutions, for which isothermal amplification is well suited. Recombinase polymerase amplification (RPA) is one the most studied isothermal alternatives, and here we provide two manuscripts addressing RPA applications. A practical example of an RPA application to a relevant clinical pathogen is provided by Liu et al. They have devised an RPA methodology to detect *Pseudomonas aeruginosa*, a pathogen involved in a wide range of clinical infections. They have combined RPA with a sequence-specific CRISPR-Cas biosensing system and, after optimization, the solution was validated in clinical samples. It was able to detect as low as 60 fg (~8 copies) of *P. aeruginosa* genomic DNA per reaction, no cross-reactivity was observed with 17 other species/strains. Performance on clinical samples was evaluated in 96 clinical samples, by comparison with a microfluidic chip. All results (19 positive and 77 negative for *P. aeruginosa*) were consistent with the microfluidic method.

The manuscript by Tan et al. reviews the principle and the different variations of RPA techniques, and it further provides a comparison with other amplification-based strategies. Clearly this technique holds great promise for future deployable solutions, with very short reaction times (5 to 20 minutes) and simple protocols and primers design. Further improvements on reagents availability and overall sensitivity, will certainly take these techniques to the next level of development.

All these works clearly show that the array of molecular techniques available today have the potential to improve, guide and expedite diagnosis, increasing the odds of a successful treatment and saving costs and, eventually, human lives. While, this Research Topic of articles showcases a limited diversity of molecular techniques/technologies that are today available in most of the research and routine laboratories; many more examples could be highlighted, with equal value and with great potential of application. Molecular diagnostics are reshaping our understanding of the microbial world and these new emerging alternatives are pushing the boundaries on our ability to quickly detect microorganisms with high accuracy. This research field will certainly bring exciting advances in the next years for diagnostic laboratories.

## Author contributions

CA: Writing – review & editing, Writing – original draft. LC: Writing – review & editing, Writing – original draft.

